# Generalizability of findings from neurobiological studies of individuals with first-episode psychosis: a cohort study

**DOI:** 10.1038/s41537-026-00762-x

**Published:** 2026-05-09

**Authors:** Alexis E. Cullen, Maria Lee, Pontus Josefsson, Carl M. Sellgren, Sophie Erhardt, Ellenor Mittendorfer-Rutz, Simon Cervenka

**Affiliations:** 1 Division of Insurance Medicine, Department of Clinical Neuroscience, Karolinska Institutet, Stockholm, Sweden; 2 Department of Psychosis Studies, Institute of Psychiatry, Psychology & Neuroscience, King’s College London, London, UK; 3 Centre for Psychiatry Research, Department of Clinical Neuroscience, Karolinska Institutet, & Stockholm Health Care Services, Region Stockholm, Stockholm, Sweden; 4 Department of Physiology and Pharmacology, Karolinska Institutet, Stockholm, Sweden; 5 Department of Medical Sciences, Psychosis Research and Preventive Psychiatry, Uppsala University, Uppsala, Sweden

**Keywords:** Psychosis, Biomarkers

## Abstract

Using Swedish register data, we estimated the proportion of individuals with first-episode psychosis (FEP) who would be eligible to participate in a typical neurobiological study and found that only 39% met criteria. Eligible and ineligible individuals differed on sociodemographic variables and two-year outcome measures (inpatient admission, work disability). Our findings suggest that neurobiological studies may yield samples that are not fully representative of the wider FEP population, potentially limiting generalisability.

## Introduction

Neurobiological studies of individuals with psychosis have the potential to elucidate disease mechanisms, identify new therapeutic targets, and support precision psychiatry approaches^1,2^. To minimise the effects of long-term illness, including pharmacological treatment exposure, such studies typically recruit antipsychotic-naïve patients experiencing their first episode of psychosis (FEP) who fulfil stringent eligibility criteria and who are willing and able to complete extensive assessments. However, these restrictions may yield unrepresentative samples, thereby limiting generalisability^3^. Using data from the nationwide Swedish registers and the Karolinska Schizophrenia Project (KaSP)—an ongoing, neurobiological study of individuals with FEP^4,5^—we aimed to determine the proportion of individuals with FEP in the patient registers who met KaSP eligibility criteria and compare the characteristics and outcomes of register-based eligible and ineligible FEP cases with those of KaSP participants.

## Methods

The Regional Ethics Board of Stockholm provided approval for the register-based study (DNR: 2007/762-31 and 2024/08708-02) and KaSP (DNR: 2010/879-31-1). Our protocol was pre-registered (https://osf.io/t8wh2) and we followed the Strengthening the Reporting of Observational Studies in Epidemiology (STROBE) – cohort study guidelines (Supplementary Table 1).

### Study populations

Individuals with FEP aged 18–45 years were recruited to KaSP^4,5^ at the time of their first healthcare contact for psychosis (typically within 4 weeks) via psychiatric clinics in Stockholm County and met diagnostic criteria for a primary psychotic disorder^6^, namely, schizophrenia, schizophreniform disorder, schizoaffective disorder, delusional disorder, brief psychotic disorder, or psychotic disorder not otherwise specified, using the Structured Clinical Interview for DSM-IV (SCID)^7^. Exclusion criteria were: (i) exposure to antipsychotic medication >4 weeks prior to recruitment, (ii) current/lifetime substance abuse, (iii) any severe somatic illness or neurological disorder, (iv) intellectual disabilities, (v) autism spectrum disorder, and (vi) serious head injuries. Participants provided written informed consent. This study used data from participants recruited from 2011 to 2021, for whom register data were available through December 2021 (*N* = 94)—to ensure equal follow-up, outcomes were assessed during the two years after inclusion.

The register-based (RB) cohort was derived using the nationwide Swedish healthcare and social insurance registers (Supplementary Table 2). According to current Swedish regulations, the use of national data for research does not require informed consent from individuals. The RB cohort included individuals aged 18–45 years, who received their first main primary psychotic disorder diagnosis (International Classification of Diseases–version 10 [ICD-10]^8^ codes: F20, F22, F23, F25, F28, or F29) in inpatient/specialist outpatient services during 2011-2021, and lived in Stockholm County at the time of diagnosis. To capture first-episode cases, individuals were required to have no previous main or side diagnoses of broadly-defined psychotic disorders (ICD-10: F20-F29) in the three years prior to their FEP diagnosis date. To account for the time delay between first contact date and recruitment of KaSP participants, for register-based cases, we set an artificial ‘inclusion date’ at 14 days after the first diagnosis of psychotic disorder and required that individuals remained alive on this date. Cohort members were then categorised as potentially eligible vs. ineligible to participate in KaSP (detailed in Supplementary Table 3). In brief, individuals with recorded purchases of antipsychotic medications >4 weeks prior to inclusion date, any main or side diagnoses of substance use disorder, intellectual disability, autism spectrum disorder, or head/intracranial injuries in inpatient or specialist outpatient care in the three previous years, or any main diagnoses of severe somatic conditions or neurological disorders in inpatient care in the three years prior to inclusion, were ineligible. All RB individuals were then followed in the registers for two years or until death/emigration. Individuals whose diagnoses changed during the study period (i.e., from a primary psychotic disorder to another psychiatric disorder) were not excluded from the register-based cohort or KaSP.

### Sociodemographic variables and outcomes

Definitions and data sources are detailed in Supplementary Table 4. Sociodemographic variables (age, sex, country of birth, family situation, level of education) were obtained from registers or self-reported by KaSP participants. In both cohorts, register data were used to determine outcomes during the 2-year follow-up, including (i) inpatient admissions for broadly-defined psychotic disorder (ICD-10 codes: F20-F29), (ii) inpatient admissions for any psychiatric disorder or attempted suicide (ICD-10 codes: F00-F99, X60-X84, Y10-Y34), and (iii) work disability (net sickness absence and disability pension days). For each outcome, we derived binary variables (none vs. any) and, among those who experienced at least one event, the total number of days.

### Analyses

Analyses were conducted using Stata version 19.5. We first determined the proportion of RB-All individuals who would be potentially eligible to participate in KaSP. Sociodemographic and outcome variables were then summarised using means, proportions, and incidence rates (per person-year) for all groups (RB-All vs. RB-Eligible vs. RB-Ineligible vs. KaSP) with group differences (*α* < 0.05) determined using immediate commands.

## Results

### Application of eligibility criteria

In total, 4957 individuals (mean [SD] age, 30.8 [7.8] years; 3060 [61.7%] men) residing in Stockholm County were diagnosed with FEP from 2011 to 2021, of whom 1942 (39.2%) met KaSP eligibility criteria and 3015 (60.8%) were ineligible. Antipsychotic medication exposure >4 weeks prior to inclusion and substance use disorder history were the most common exclusion criteria, detected in 38.0% and 30.3% of the RB-All cohort, respectively (Supplementary Fig. 1). All other exclusion criteria were prevalent in ≤7% and commonly occurred in combination with antipsychotic medication exposure and substance use disorder.

### Cohort characteristics

Compared to the RB-Ineligible group, RB-Eligible individuals were more likely to be women, born outside of Sweden, married/cohabiting with children, to have completed university education, and to have brief/transient psychotic disorder (Table 1). KaSP participants were younger on average and showed marked differences in FEP diagnoses relative to both RB groups: Notably, schizophrenia was highly prevalent in the KaSP group but accounted for only a small proportion of the RB-Eligible and RB-Ineligible groups (54.6%, 3.8%, and 7.4%, respectively) whereas brief/transient psychotic disorder showed the reverse pattern (present among 5.3%, 30.2%, and 23.5%, respectively). KaSP participants were also less likely to be married/cohabiting and living with children than RB-Eligible cases.

**Table 1:** Characteristics of study cohorts at baseline.

Characteristic	RB-All (*N* = 4957)	RB-Eligible (*N* = 1942)	RB-Ineligible (*N* = 3015)	KaSP (*N* = 94)	Statistical comparison
Age, mean (SD) years	30.8 (7.8)	31.0 (7.6)	30.7 (7.9)	27.6 (6.5)	^a^ t = 3.95***, ^b^ t = 4.26***, ^c^ t = 3.76***, ^d^ t = 1.32
Sex, *n* (%)					
Women	1897 (38.3)	859 (44.2)	1038 (34.4)	38 (40.4)	^a^ *χ*^*2*^ = 0.18, ^b^ *χ*^*2*^ = 0.53, ^c^ *χ*^*2*^ = 1.45, ^d^ *χ*^*2*^ = 48.07***
Men	3060 (61.7)	1083 (55.8)	1977 (65.6)	56 (59.6)	
Country of birth, *n* (%)					
Sweden	3430 (69.2)	1274 (65.6)	2156 (71.5)	70 (74.5)	^a^ *χ*^*2*^ = 1.21, ^b^ *χ*^*2*^ = 3.14, ^c^ *χ*^*2*^ = 0.39, ^d^ *χ*^*2*^ = 19.33***
Other	1527 (30.8)	668 (34.4)	859 (28.5)	24 (25.5)	
Family situation, *n* (%)					
Married or cohabitant with children	534 (10.8)	298 (15.3)	236 (7.8)	6 (6.4)	^a^ *χ*^*2*^ = 1.86, ^b^ *χ*^*2*^ = 5.67*, ^c^ *χ*^*2*^ = 0.27, ^d^ *χ*^*2*^ = 69.45***
Other	4423 (89.2)	1644 (84.7)	2779 (92.2)	88 (93.6)	
Educational level, *n* (%)					
Compulsory (0–9 years)	1652 (33.3)	466 (24.0)	1186 (39.3)	26 (27.7)	^a^ *χ*^*2*^ = 3.53, ^b^ *χ*^*2*^ = 0.66, ^c^ *χ*^*2*^ = 12.98**, ^d^ *χ*^*2*^ = 198.85***
High school (10–12 years)	1984 (40.0)	768 (39.5)	1216 (40.3)	35 (37.2)	
University ( > 12 years)	1321 (26.6)	708 (36.5)	613 (20.3)	33 (35.1)	
Diagnosis, *n* (%)					
Schizophrenia	298 (6.0)	74 (3.8)	224 (7.4)	53 (56.4)	^a^ FE***, ^b^ FE***, ^c^ FE***, ^d^ *χ*^*2*^ = 105.87***
Persistent delusional disorder	438 (8.8)	193 (9.9)	245 (8.1)	4 (4.3)	
Brief/transient psychotic disorder	1296 (26.1)	587 (30.2)	709 (23.5)	5 (5.3)	
Schizoaffective disorder	179 (3.6)	26 (1.3)	153 (5.1)	3 (3.2)	
Other non-organic psychotic disorder	61 (1.2)	12 (0.6)	49 (1.6)	0 (0)	
Unspecified non-organic psychotic disorder	2685 (54.2)	1050 (54.1)	1635 (54.2)	29 (30.9)	

*RB* Register-based, *KaSP* Karolinska Schizophrenia Project, *t* t statistic, *χ*^*2*^ chi-squared statistic, *FE* Fisher’s exact.

*p < 0.05, **p < 0.01, ***p < 0.001.

^a^RB-All vs. KaSP.

^b^RB-Eligible vs. KaSP.

^c^RB-Ineligible vs. KaSP.

^d^RB-Eligible vs. RB-Ineligible.

### Clinical and functional outcomes

**Fig. 1:**
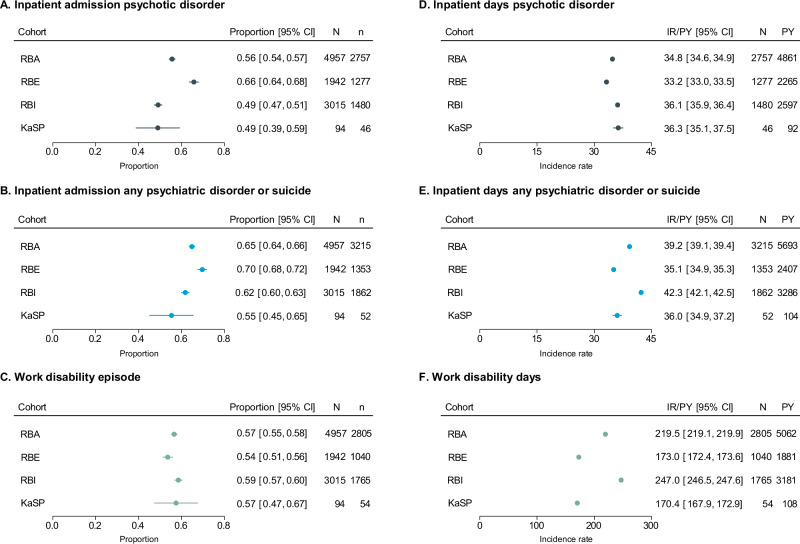
Clinical and functional outcomes during the 2-year follow-up presented for all study cohorts. Binary outcomes are presented in panels **A**, **B**, and **C** as the proportion of individuals within each cohort who experienced the outcome at least once during the follow-up. Count outcomes (number of days) are presented in panels **D**, **E**, and **F** as the incidence rate per person-year among individuals who experienced the outcome during the 2-year follow-up. RBA register-based all; RBE register-based eligible; RBI register-based ineligible; KaSP Karolinska Schizophrenia Project; N total number of individuals; n number of individuals experiencing outcome; CI confidence interval; IR incidence rate; PY person-years.

RB-Eligible individuals were significantly more likely to be hospitalised for broadly-defined psychotic disorder and for any psychiatric disorder/suicide attempt when compared to both the RB-Ineligible and KaSP cohorts but were somewhat less likely have an episode of work disability than the RB-Ineligible group (Fig. 1 and Supplementary Table 5). Among those who experienced these outcomes, the RB-Eligible group had significantly fewer inpatient days for broadly-defined psychotic disorder and any psychiatric disorders/suicide attempt over the 2-year follow-up and fewer work disability days compared to RB-Ineligible individuals. In addition, the KaSP group spent more days in hospital for psychotic disorder than the RB-Eligible group but had fewer inpatient days for any psychiatric disorder/suicide attempt and fewer work disability days when compared RB-Ineligible individuals.

## Discussion

We observed that only 39% of individuals with FEP would be eligible to participate in a typical neurobiological study, with the most common exclusion reasons being antipsychotic use >4 weeks before inclusion and substance use disorder. These findings illustrate how standard eligibility criteria may yield samples that differ from the wider FEP population. Consistent with these results, a recent study which used Swedish register data to investigate the proportion of individuals with primary psychotic disorders in real-world samples who would be potentially eligible to participate in randomised controlled trials (RCTs) of pharmacotherapies found that less than 22% would meet standard eligibility criteria^9^, which is markedly lower than the proportion of individuals in Sweden with major depressive disorder who were determined to be potentially eligible to participate in RCTs (65%)^10^. Together, these findings suggest that results from neurobiological studies and pharmacological trials of individuals with FEP may have limited generalisability to the wider population of individuals with these disorders.

KaSP participants and register-based groups (eligible and ineligible) differed on sociodemographic characteristics and diagnostic profile. Register-based groups were older on average than KaSP participants, which suggests that younger individuals might be more motivated or willing to participate in neurobiological studies. However, the mean age at FEP onset among register-based individuals was also higher than we have observed in previous investigations using the nationwide Swedish registers^11^. This difference may be due to the fact that our previous study included individuals aged 18-35 years (vs. 18–45 years in the present study) and that the present study was restricted to people living in Stockholm, a region that experienced a negative net migration effect among individuals aged 18-22 years during the study period^12^. The higher prevalence of schizophrenia diagnoses among KaSP participants compared to register-based cases likely reflects differences between diagnoses derived in research vs. real-world settings and possibly a reluctance on behalf of clinicians to assign this diagnosis in practice due to stigma.

RB-Eligible and RB-Ineligible individuals differed on sociodemographic factors, including sex, country of birth, education, and family situation. Our finding that eligible individuals were more likely to be hospitalised for psychiatric disorders (including psychotic disorders) and/or suicide attempts, but had fewer inpatient days than ineligible individuals, may reflect differences in clinical complexity, particularly the high prevalence of substance use disorder in the ineligible group. Such comorbidities may contribute to both higher thresholds for admission and longer inpatient stays once admitted. Similarly, higher rates and longer durations of work disability in the ineligible group may be partly explained by greater psychiatric and somatic comorbidity. The fact that KaSP participants who experienced a work disability episode had fewer disability days than ineligible register-based cases tentatively suggests that individuals who are able to participate in neurobiological studies may have less severe functional impairments.

Our study suggests that results derived from neurobiological studies, particularly those requiring that participants have minimal exposure to antipsychotic medications and free from comorbid substance use disorders, may have limited generalisability to the wider FEP population. More inclusive criteria may improve representativeness, but their usefulness depends on the specific research question. For some investigations, such as Positron Emission Tomography (PET) studies measuring dopamine receptors^13^, strict eligibility criteria remain necessary to avoid confounding effects of antipsychotic medications (which predominately target dopamine neurotransmission). However, studies focused on broader neurobiological measures or more clinically-oriented investigations may benefit from less restrictive criteria. Deriving weighted samples, using the distribution of sociodemographic variables in large (ideally national) populations, may be another way to address non-representativeness^14^, but such data are not always available.

Some limitations should be noted. The register-based eligibility criteria that we applied in this study are proxies for the criteria applied in KaSP, which were based on detailed clinical assessments. Similarly, the methods used to derive FEP diagnoses in these samples (real-world vs. researcher-derived) likely contributed to the marked differences in diagnoses that we observed across the register-based and KaSP cohorts. Although these findings may also reflect underlying differences in clinical characteristics, we were unable to assess this directly as measures of symptom/illness severity are unavailable in the registers. In addition, we were not able to examine diagnostic groups separately due to the limited sample size of the KaSP cohort. A further limitation is that the prescribed drug register does not capture medications administered in inpatient settings; however, as eligibility was determined using both diagnostic records and a three-year look-back period for antipsychotic use, this is unlikely to lead to misclassification of RB individuals as eligible vs. ineligible.

To conclude, eligibility criteria applied in typical neurobiological studies yield groups that are not fully representative of the wider FEP population in terms of sociodemographic factors and outcomes. Relaxing eligibility criteria and sample weighting are potential strategies to improve representativeness, but the extent to which it is appropriate and useful to apply these approaches is highly dependent on the research question.

## Supplementary information

Supplementary information

## Data Availability

Register data reported in this paper cannot be made publicly available due to privacy regulations. According to the General Data Protection Regulation, the Swedish law SFS 2018:218, the Swedish Data Protection Act, the Swedish Ethical Review Act, and the Public Access to Information and Secrecy Act, these types of sensitive data can only be made available for specific purposes, including research, that meets the criteria for access to this type of sensitive and confidential data as determined by a legal review. Readers may contact Professor Ellenor Mittendorfer-Rutz (ellenor.mittendorfer-rutz@ki.se) regarding the data.
