# Supplementary material for: Generalizability of findings from neurobiological studies of individuals with first-episode psychosis: a cohort study
Source: Schizophrenia (Heidelb). 2026 May 9;12(1):43. doi: 10.1038/s41537-026-00762-x (PMC13157486; doi:10.1038/s41537-026-00762-x)
Supplement: Supplementary file 1 — Supplementary information [file 41537_2026_762_MOESM1_ESM.docx]

Supplementary Material

**Cullen AE, Lee M, Josefsson P, Sellgren C, Erhardt E, Mittendorfer-Rutz E, Cervenka S. Generalizability of findings from neurobiological studies of individuals with first-episode psychosis: A cohort study.**

**Supplementary Table 1.** Strengthening the Reporting of Observational Studies in Epidemiology (STROBE) Checklist for reporting observational cohort studies

|  | **Item** | **Recommendation** | **Page** |
| --- | --- | --- | --- |
| **Title and abstract** | 1 | (*a*) Indicate the study’s design with a commonly used term in the title or the abstract | 1, 3 |
|  |  | (*b*) Provide in the abstract an informative and balanced summary of what was done and what was found | 3 |
| Introduction | | | |
| Background/rationale | 2 | Explain the scientific background and rationale for the investigation being reported | 4 |
| Objectives | 3 | State specific objectives, including any prespecified hypotheses | 4 |
| Methods | | | |
| Study design | 4 | Present key elements of study design early in the paper | 4 |
| Setting | 5 | Describe the setting, locations, and relevant dates, including periods of recruitment, exposure, follow-up, and data collection | 4, 5 |
| Participants | 6 | (*a*) Give the eligibility criteria, and the sources and methods of selection of participants. Describe methods of follow-up | SM |
|  |  | (*b*) For matched studies, give matching criteria and number of exposed and unexposed | N/A |
| Variables | 7 | Clearly define all outcomes, exposures, predictors, potential confounders, and effect modifiers. Give diagnostic criteria, if applicable | SM |
| Data sources/ measurement | 8* | For each variable of interest, give sources of data and details of methods of assessment (measurement). Describe comparability of assessment methods if there is more than one group | SM |
| Bias | 9 | Describe any efforts to address potential sources of bias | SM |
| Study size | 10 | Explain how the study size was arrived at | N/A |
| Quantitative variables | 11 | Explain how quantitative variables were handled in the analyses. If applicable, describe which groupings were chosen and why | SM |
| Statistical methods | 12 | (*a*) Describe all statistical methods, including those used to control for confounding | 5 |
|  |  | (*b*) Describe any methods used to examine subgroups and interactions | 5 |
|  |  | (*c*) Explain how missing data were addressed | NA |
|  |  | (*d*) If applicable, explain how loss to follow-up was addressed | 5 |
|  |  | (*e*) Describe any sensitivity analyses | NA |
| Results | | |  |
| Participants | 13* | (a) Report numbers of individuals at each stage of study— e.g., numbers potentially eligible, examined for eligibility, confirmed eligible, included in the study, completing follow-up, and analysed | 5, Fig 1 |
|  |  | (b) Give reasons for non-participation at each stage | NA |
|  |  | (c) Consider use of a flow diagram | NA |
| Descriptive data | 14* | (a) Give characteristics of study participants (e.g., demographic, clinical, social) and information on exposures and potential confounders | 6 |
|  |  | (b) Indicate number of participants with missing data for each variable of interest | NA |
|  |  | (c) Summarise follow-up time (e.g., average and total amount) | Fig 2 |
| Outcome data | 15* | Report numbers of outcome events or summary measures over time | Fig 2 |

SM, Supplementary Material.

**Supplementary Table 2.** Swedish Healthcare and Social Insurance Registers

| **Database** | **Description** |
| --- | --- |
| Total Population Register (TPR) | Maintained by Statistics Sweden, providing basic demographic information (including region of residence for the entire population, available from 1968.^1^ |
| National Patient Register (NPR) | Maintained by the National Board of Health and Welfare, providing dates and diagnoses associated with inpatient care (available from 1987) and specialist outpatient care (available from 2001) covering both psychiatric and somatic healthcare services.^2,3^ |
| Longitudinal Integrated Database for Health Insurance and Labour Market Studies (LISA) | Maintained by Statistics Sweden, providing detailed sociodemographic data, including unemployment days and type of residence region for all individuals aged ≥16 years since 1990.^4^ |
| Micro-Data for Analyses of Social Insurance (MiDAS) | Detailing sickness absence and disability pension payments covered by the Swedish Social Insurance Agency, available from 1994.^5^ |
| Prescribed Drug Register (PDR) | Administered by the National Board of Health and Welfare, capturing all prescribed and dispensed medications in Swedish pharmacies (except those administered in hospital), available from July 2005.^6^ |
| Cause of Death Register (CDR) | Administered by the National Board of Health and Welfare, providing information on date during the study period, available from 1952.^7^ |

**Supplementary Table 3.** Procedures for defining the register-based first-episode psychosis cohort and applying eligibility criteria

| **Step** | **Definition** | **Register** |
| --- | --- | --- |
| 1 | Received a main diagnosis of psychotic disorder (ICD-10 codes: F20, F22, F23, F25, F28, F29) in inpatient/specialised outpatient care between 1st of January 2011 and 31^st^ of December 2021, inclusive, when aged 18-45 years ^a^ | NPR |
| 2 | Registered as a resident of Sweden in the year of diagnosis and 3 calendar years prior to diagnosis (to ensure all individuals had a ‘look back’ period to check the remaining steps) | LISA |
| 3 | No previous main or side diagnoses of broadly-defined psychotic disorder (ICD-10 codes: F20-F29) in inpatient/specialised outpatient care in the 3 years prior to first observed diagnosis in order minimise risk of misclassifying individuals as first-episode cases | NPR |
| 4 | Not registered as having died before the inclusion date (i.e., 14 days after diagnosis) to account for the time delay (1-4 weeks) between first contact and recruitment of KaSP participants | CDR |
| 5 | No recorded purchases of antipsychotic medication (Anatomic Therapeutic Chemical classification [ATC] codes N05A, omitting N05AA02, N05AB01, N05AB04, N05AD03, N05AD08, N05AN01) more than 4 weeks (28 days) before inclusion date (with a look back window of 3 years) | PDR |
| 5 | No recorded main or side diagnoses of substance use disorder (ICD-10 codes: F10-F19) in inpatient/specialised outpatient care in the three years before inclusion date | NPR |
| 5 | No previous diagnosis of intellectual disability (ICD-10 codes: F70-F79) as a main or side diagnosis in inpatient/specialist outpatient care within 3 years before inclusion | NPR |
| 5 | No previous diagnosis of autism spectrum disorder (ICD-10 codes: F84) as a main or side diagnosis in inpatient/specialist outpatient care in the 3 years before inclusion | NPR |
| 5^a^ | No inpatient care for any severe somatic conditions, including infections, neoplasms, endocrine/nutritional/metabolic diseases, and disorders of the circulatory, respiratory, or digestive systems (ICD-10 codes: A00-B99, C00-D48, E00-E90, I00-I99, J00-J99, K00-K93), recorded as main diagnosis, in the 3 years prior to inclusion | NPR |
| 5^a^ | No inpatient care for any neurological disorders (ICD-10 codes: G00-G99), where these are main diagnoses, in the 3 years prior to inclusion | NPR |
| 5 | No inpatient/specialised outpatient care for head injuries, intracranial injuries, crushing injuries to the head, or traumatic amputation of part of the head (ICD-10 codes: S02.0, S02.1, S02.3, S02.4, S02.6, S02.7, S02.8, S02.9, S06, S07, S08), where these are main or side diagnoses, in the 3 years prior to inclusion | NPR |

NPR, National Patient Register; CDR, Cause of Death Register; PDR, Prescribed Drug Register. ^a^ Inclusion date set at 14 days after the first diagnosis.

^a^ To align the registry-based eligibility criteria with the KaSP recruitment procedure, previous somatic and neurological conditions were only considered exclusionary if recorded as main diagnoses in inpatient settings, as these were more likely to reflect clinically significant conditions.

**Supplementary Table 4.** Definitions and Data Sources for Sociodemographic Variables and 2-year Outcome Measures

| **Variable** | **Definition** | **Data source** |  |
| --- | --- | --- | --- |
|  |  | **RB cohort** | **KaSP cohort** |
| **Sociodemographic variables** |  |  |  |
| Age | Age (years) at inclusion date | LISA^a^ | Self-report |
| Sex | Categorised as men vs. women | LISA^a^ | Self-report |
| Country of birth | Categorised as Sweden vs. other | LISA^a^ | National Agency for Education |
| Family situation | Categorised as married/cohabiting with children living at home vs. other | LISA^a^ | Self-report |
| Level of education | Highest level of education completed, categorised as compulsory (≤9 years) vs. high school (10-12 years) vs. university (>12 years) | LISA^a^ | Self-report |
| Psychotic disorder diagnosis | Categorised as schizophrenia/schizophreniform disorder vs. persistent delusional disorder vs. acute and transient psychotic disorder vs. schizoaffective disorder vs. other non-organic psychotic disorder vs. psychotic disorder not otherwise specified | NPR^b^ | SCID |
| **Outcome variables at 2 years** ^c^ |  |  |  |
| Inpatient admission for psychotic disorder | Any inpatient admission within the 2-year follow-up where a main diagnosis of broadly-defined psychotic disorder (ICD-10 codes: F20-F29) was assigned at discharge, categorised as no vs. yes | NPR | NPR |
| Inpatient admission for any psychiatric disorder / suicide attempt | Any inpatient admission within the 2-year follow-up where a main diagnosis of any psychiatric disorder (ICD-10 codes: F00-F99) or attempted suicide (ICD-10 codes: X60-X84, Y10-Y34) was assigned at discharge, categorised as no vs. yes | NPR | NPR |
| Work disability | Any days of sickness absence (in spells exceeding 14 days) or receipt of disability pension within the 2-year follow-up, both measures converted to net days (number of days * extent of working time) and summed, categorised as no vs. yes | MiDAS | MiDAS |
| Length of stay for psychotic disorder | Total number of inpatient days attributable to broadly-defined psychotic disorder (ICD-10 codes: F20-F29) among those who were hospitalised at least once for this outcome, expressed as rate (number of days per person-year) | NPR^c^ | NPR^c^ |
| Length of stay for any psychiatric disorder / suicide attempt | Total number of inpatient days attributable to any psychiatric disorder (ICD-10 codes: F00-F99) or attempted suicide (ICD-10 codes: X60-X84, Y10-Y34) among those who were hospitalised at least once for this outcome, expressed as rate (number of days per person-year) | NPR^c^ | NPR^c^ |
| Length of work disability | Sum of net days (number of days * extent of working time) for sickness absence and disability pension among those who experienced any days, expressed as rate (number of days per person-year) | MiDAS | MiDAS |

KaSP, Karolinska Schizophrenia Project; LISA, Longitudinal Integrated Database for Health Insurance and Labour Market Studies; MiDAS, Micro-Data for Analyses of Social Insurance; NPR, National Patient Register; PDR, Prescribed Drug Register; RB, register-based.

1. LISA variables measured on the 31^st^ of December on the calendar year prior to inclusion date.
2. For individuals in the register-based cohort, we obtained all relevant psychotic disorder diagnoses recorded between the first diagnosis date and inclusion date and used a hierarchy to determine primary diagnosis where more severe/chronic conditions were prioritised as follows: F20 > F25 > F22 > F23 > F28 > F29.
3. Outcomes were measured from inclusion date to 730 days post-inclusion except for inpatient days among individuals whose first admission for a primary psychotic disorder commenced before the inclusion date – for these individuals, we counted inpatient days from the start of the admission and individuals were followed for 730 days from this date.

**
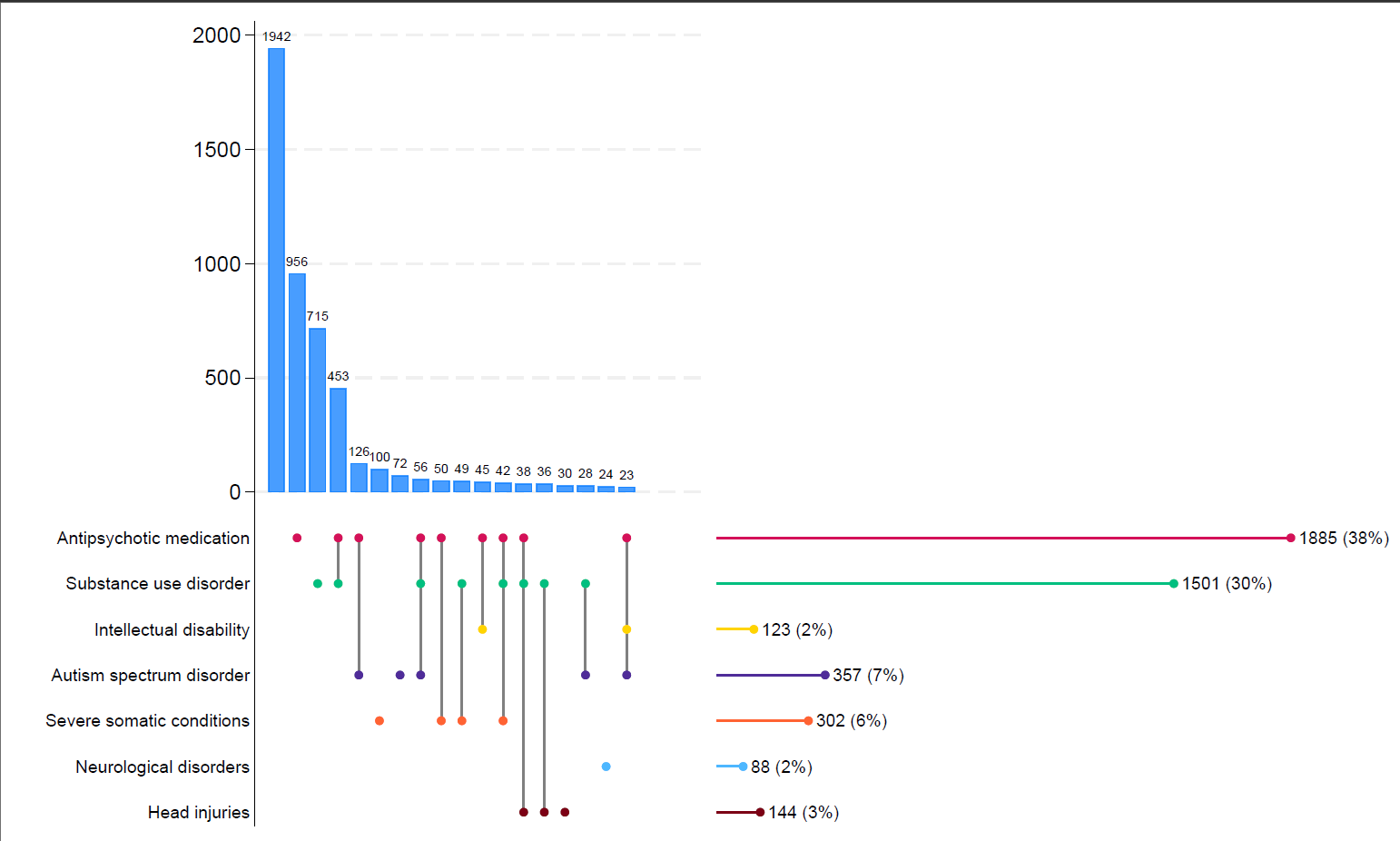
**

**Supplementary Figure 1.** Upset plot showing number (%) of individuals within the register-based first-episode psychosis cohort who meet each eligibility criterion and their combinations (shown for combinations with ≥20 individuals).

**Supplementary Table 5.** Statistical Comparison of Binary and Count Outcome Variables

| **Outcome** | **RB-All**  **(N = 4957)** | **RB-Eligible**  **(N = 1942)** | **RB-Ineligible**  **(N = 3015)** | **KaSP**  **(N = 94)** | **Statistical comparison** |
| --- | --- | --- | --- | --- | --- |
| Binary outcomes, Proportion (95% CI) |  |  |  |  |  |
| Admission psychotic disorder | 0.56  (0.54, 0.57) | 0.66  (0.64, 0.68) | 0.49  (0.47, 0.51) | 0.49  (0.38, 0.59) | ^a^ z = 1.29, ^b^ z = 3.34***, ^c^ z = 0.03, ^d^ z = 11.53*** |
| Admission any psychiatric disorder or suicide | 0.65  (0.64, 0.66) | 0.70  (0.68, 0.72) | 0.62  (0.60, 0.63) | 0.55  (0.45, 0.66) | ^a^ z = 1.92, ^b^ z = 2.94**, ^c^ z = 1.26, ^d^ z = 5.70*** |
| Work disability episode | 0.57  (0.55, 0.58) | 0.54  (0.51, 0.56) | 0.59  (0.57, 0.60) | 0.57  (0.47, 0.68) | ^a^ z = -0.17, ^b^ z = -0.74, ^c^ z = 0.21, ^d^ z = -3.46*** |
| Count outcomes, IR/PY (95% CI) |  |  |  |  |  |
| Inpatient days for psychotic disorder | 34.8  (34.6, 34.9) | 33.2  (33.0, 33.5) | 36.1  (35.9, 36.4) | 36.3  (35.1, 37.5) | ^a^ IRD = 1.52*, ^b^ IRD = 3.08***, ^c^ IRD = 0.16,  ^d^ IRD = 2.92*** |
| Inpatient days any psychiatric disorder or suicide | 39.2  (39.1, 39.4) | 35.1  (34.9, 35.3) | 42.3  (42.1, 42.5) | 36.0  (34.9, 37.2) | ^a^ IRD = -3.21***, ^b^ IRD = 0.95, ^c^ IRD = -6.25***,  ^d^ IRD = 7.20*** |
| Work disability days | 219.5  (219.1, 219.9) | 173.0  (172.4, 173.6) | 247.0  (246.5, 247.6) | 170.4  (168.0, 172.9) | ^a^ IRD = -49.13***, ^b^ IRD = -2.59,  ^c^ IRD = -76.64***, ^d^ IRD = 74.06*** |

RB, Register-based; KaSP, Karolinska Schizophrenia Project; CI, confidence interval; z, statistic directed from two-sample test of proportions; IRD, incidence rate difference where p values calculated using exact method. Binary outcomes (proportions) are calculated by cohort for all individuals; count outcomes (incidence rate per person-year) are calculated by cohort for those individuals who experienced at least one outcome event. ^a^ RB-All (reference group) vs. KaSP, ^b^ RB-Eligible (reference group) vs. KaSP, ^c^ RB-Ineligible (reference group) vs. KaSP, ^d^ RB-Eligible (reference group) vs. RB-Ineligible, * p<0.05, ** p<0.01, *** p<0.001

**REFERENCES**

1 Ludvigsson, J. F. *et al.* Registers of the Swedish total population and their use in medical research. *Eur J Epidemiol* **31**, 125-136 (2016).

2 National Board of Health and Welfare. (Stockholm, 2016).

3 Ludvigsson, J. F. *et al.* External review and validation of the Swedish national inpatient register. *BMC Public Health* **11**, 450 (2011).

4 Ludvigsson, J. F., Svedberg, P., Olen, O., Bruze, G. & Neovius, M. The longitudinal integrated database for health insurance and labour market studies (LISA) and its use in medical research. *Eur J Epidemiol* **34**, 423-437 (2019).

5 Forsakringskassan. (Stockholm, 2011).

6 Wallerstedt, S. M., Wettermark, B. & Hoffmann, M. The First Decade with the Swedish Prescribed Drug Register - A Systematic Review of the Output in the Scientific Literature. *Basic Clin Pharmacol Toxicol* **119**, 464-469 (2016).

7 Brooke, H. L. *et al.* The Swedish cause of death register. *Eur J Epidemiol* **32**, 765-773 (2017).
